# Reviewing deaths in British and US hospitals: a study of two scales for assessing preventability

**DOI:** 10.1136/bmjqs-2015-004849

**Published:** 2016-06-22

**Authors:** Semira Manaseki-Holland, Richard J Lilford, Jonathan R B Bishop, Alan J Girling, Yen-Fu Chen, Peter J Chilton, Timothy P Hofer, The UK Case Note Review Group

**Affiliations:** 1 Public Health, Epidemiology and Biostatistics, University of Birmingham, Birmingham, UK; 2 Warwick Medical School, University of Warwick, Coventry, UK; 3 Warwick Business School, University of Warwick, Coventry, UK; 4 Center for Clinical Management Research, VA Ann Arbor Healthcare System, Ann Arbor, Michigan, USA; 5 Department of Medicine, University of Michigan Medical School, Ann Arbor, Michigan, USA

**Keywords:** Mortality (standardized mortality ratios), Quality improvement methodologies, Adverse events, epidemiology and detection, Medical error, measurement/epidemiology

## Abstract

**Background:**

Standardised mortality ratios do not provide accurate measures of preventable mortality. This has generated interest in using case notes to assess the preventable component of mortality. But, different methods of measurement have not been compared. We compared the reliability of two scales for assessing preventability and the correspondence between them.

**Methods:**

Medical specialists reviewed case notes of patients who had died in hospital, using two instruments: a five-point Likert scale and a continuous (0–100) scale of preventability. To enhance generalisability, we used two different hospital datasets with different types of acute medical patients across different epochs, and in two jurisdictions (UK and USA). We investigated the reliability of measurement and correspondence of preventability estimates across the two scales. Ordinal mixed effects regression methods were used to analyse the Likert scale and to calibrate it against the continuous scale. We report the estimates of the probability a death could have been prevented, accounting for reviewer inconsistency.

**Results:**

Correspondence between the two scales was strong; the Likert categories explained most of the variation (76% UK, 73% USA) in the continuous scale. Measurement reliability was low, but similar across the two instruments in each dataset (intraclass correlation: 0.27, UK; 0.23, USA). Adjusting for the inconsistency of reviewer judgements reduced the proportion of cases with high preventability, such that the proportion of all deaths judged probably or definitely preventable on the balance of probability was less than 1%.

**Conclusions:**

The correspondence is high between a Likert and a continuous scale, although the low reliability of both would suggest careful measurement design would be needed to use either scale. Few to no cases are above the threshold when using a balance of probability approach to determining a preventable death, and in any case, there is little evidence supporting anything more than an ordinal correspondence between these reviewer estimates of probability and the true probability. Thus, it would be more defensible to use them as an ordinal measure of the quality of care received by patients who died in the hospital.

## Introduction

Quality control and improvement strategies have often focused on death rates—including overall hospital death rates, as an indisputably severe consequence of poor care.^1^ Yet, a large body of work suggests that the signal (preventable deaths) is likely to be buried in the noise (inevitable deaths), even after risk adjustment.^2^ When overall deaths (measured in standardised mortality ratios) are used to assess healthcare quality, they are only functioning as a crude proxy for the preventable component. The lack of specificity and sensitivity^3–8^ raises the question—why not directly measure only the preventable death rates? A number of studies have now been conducted to directly measure preventable death rates in the USA,^9^ ^10^ the UK,^11^ and in continental Europe.^12^ In fact, the English National Health Service (NHS) have recently announced a policy to examine hospital deaths routinely and classify them as preventable or not using the methods from these studies.^13^

Direct measurement most commonly involves the review of each death by an expert, typically by scrutinising charts (case notes) to determine if it was preventable. Most studies define deaths as preventable or not on the balance of probabilities, as in a case of tort which requires the probability of causation to exceed 50% in order for the exposure to be considered a contributory cause of death.^14^ The assessment is based on a measurement scale that asks the reviewers to estimate the probability that the death could have been prevented. There are two candidates for such a measurement scale: a Likert scale incorporating the ‘more likely than not’ standard among the anchors or a more fine-grained continuous scale of probabilities—0–100.

Virtually, all published works on preventable deaths have used the Likert scale. We know of no papers that have measured the probability of preventable deaths on a continuous (0–100) scale, or which have investigated the correspondence of the Likert scale measurement to the underlying continuous probability construct supposedly captured by the Likert scale. The purpose of this study is to compare both the correspondence and the relative reliability of measurements obtained using the different scales.

If, as expected, the two scales are highly correlated, it would provide some degree of construct validity, by demonstrating that these two measures are related, as one would expect based on theory. Nevertheless, one scale would be preferred if it is more reliable than the other. Finally, having characterised the reliability of the scales, we can illustrate the distribution of the preventability assessments in the absence of reviewer effects and measurement noise. This removes variability in the measurement due to systematic differences across cases in the overall severity of a reviewer and the noise resulting from the inconsistency of multiple reviewers looking at a specific case.

Using two existing datasets (not created specifically for this study) where case notes had been reviewed by multiple reviewers, and where each reviewer estimated preventability on both Likert and continuous scales, our objectives are to present the following:

1. The correspondence between the two scales and the more likely than not cut-off point.
2. The reliability of each scale—the extent to which different reviews result in the same assessment.
3. The impact of low reliability on individual and population measurement of preventable deaths.
4. And, in the discussion, some implications and recommendations for the design of a programme that might use case note review to assess the preventability of deaths in a health system.

## Methods

### UK study, 2009

#### Background

Data were obtained from deaths reviewed as part of the Safer Patients Initiative (SPI), an evaluation of a large intervention to improve the quality of care in UK hospitals between 2003 and 2009. The study and its evaluation involved 22 hospitals in England and Wales, and details are published elsewhere.^15^ ^16^

#### Case notes

Case notes of 191 deceased patients admitted for respiratory complaints and over the age of 65 years were anonymised and scanned. These were then independently scrutinised by 22 reviewers in a separate exercise not previously reported in the above publications. The case notes were randomly assigned to at least two trained reviewers who used a pro forma to guide an implicit review (see below) of the notes. Each case note was reviewed by between three and seven reviewers in order to ascertain the reviewer effect on reliability. In total, 653 reviews were carried out.

#### Selection of the reviewers

Reviewers (n=22) were experienced, qualified, working clinicians (consultants in Medicine and Intensive Care Units) who were trained as described below.

### US Lab Indicators Study, 1997

#### Background

This second dataset was a de-identified subset of data originally collected as part of a study done in the late 1990s (referred to as the ‘Lab Indicators Study’), the primary goal of which was to develop quality screens based on hospital-acquired metabolic derangements and drug toxicity, as assessed by laboratory testing records. All deaths occurring in the much larger Lab Indicators Study population, along with a sample of deaths from a control population, were included in this substudy on reviewer assessments of potentially preventable mortality. This dataset thus effectively oversampled for deaths in which potentially preventable adverse events occurred. The sample was drawn from seven hospitals in the Veterans Affairs system, which at that time had one of the most advanced electronic clinical database systems in the USA, thus considerably easing the implementation of the study protocol. Details of the study are published in prior publications, including analyses presenting estimates of preventable mortality, based on the Likert scale,^10^ ^17^ but a comparative analysis of the continuous and Likert scale has not been published.

#### Case notes

Case notes of 179 deaths were reviewed, with all reviews done and data entered in the late 1990s. Of the initial sample, 66 cases (37%) were excluded by an initial review that identified and removed admissions for comfort care or palliative care and all advanced cancers, along with two additional cases in which the discharge record coding was incorrect and death had not actually occurred during the inpatient stay, leaving 111 case notes. The number of reviews per case note varied, ranging from 1 to 14, with a total of 383 reviews. Of the 111 deaths that were reviewed, 35 cases were reviewed as part of a substudy in the original project, with a target of 4–14 reviews per record. The remaining 88 records had a target of two reviews per record. Reviewers took records from randomly ordered lists of the much larger number of records targeted for review by the parent study and provided at each session to each reviewer. Because of the challenges of scheduling clinically active reviewers outside of their hospital responsibilities, the availability of charts being reviewed by other reviewers and the lack of stratification by death in assigning reviews, considerable variability in the number of reviews per record resulted in this subsample of deaths.

#### Selection of the reviewers

Thirteen practicing physicians, actively engaged in hospital medical practice, were recruited and trained in the use of the instrument (see below).

### Both studies

#### Pro forma for data extraction

Each case note was reviewed using a semistructured implicit (holistic) review (see online supplementary appendix 1).^18^ The UK study used a pro forma adapted from the US study,^10^ which was itself based on the original instrument developed by RAND for the diagnosis-related group pre-post study in 1989.^19^ The UK pro forma included a number of questions not included in the US pro forma, covering the diagnosis and cause of death. The US pro forma did not include this information, but did elicit information about whether or not a ‘do not resuscitate’ order had been placed. These data items are not used in the analysis in this paper. The US pro forma asks for a percentage estimate for the likelihood that a death could have been prevented, whereas the UK pro forma asks the reviewer to indicate this quantity on a 0–100 scale. Importantly, the modified instrument for both studies requested reviewers to classify mortality events by the level of preventability on both a Likert and a continuous scale, with similar wording for both items. The instrument guided the doctor to systematically review and evaluate different parts of the medical case note as a minimum before they came to give their overall preventability assessment.

10.1136/bmjqs-2015-004849.supp1 Supplementary appendix

In the UK study, the Likert categorisation was obtained using the question: ‘On the balance of probability (ie, >50% chance), was the death preventable? 1=Definitely Yes; 2=Probably Yes; 3=Uncertain; 4=Probably Not; 5=Definitely Not’. The percentage preventability on the continuous scale was elicited as the ‘best estimate of likelihood of preventability of death’. Similarly, but with somewhat different wording, in the US study, the Likert categorisation was obtained using the question: ‘Was patient death preventable by better quality of care? 1=Definitely Yes; 2=Probably Yes; 3=Uncertain; 4=Probably Not; 5=Definitely Not’. The percentage preventability was elicited as ‘What do you estimate the likelihood of prevention of death to be if care had been optimal?’ These specifications enabled us to do a test of ‘logical consistency’. It is clearly logically inconsistent to either: (a) choose Likert 5 (definitely not preventable) if the best preventability estimate is >50% or (b) choose Likert 1 (definitely preventable) if the best estimate is <50%.

#### Training of the reviewers

Training for the reviewers in the US study is described in prior publications, but involved reviewing sample charts and ensuring not the absence of disagreements in rating care, but rather that differences were primarily a matter of opinion, not a result of lack of understanding of the instrument or overlooking information available in different parts of the medical case note.^10^ ^20^ Training of the reviewers in the UK study mimicked that of the US study, though using a different set of exemplar case notes. All the reviewers in the UK study attended two training sessions (a full-day and a half-day), with practice case-note reviews at home in between, while the US reviewers had a single-day training. The purpose of training in both studies was therefore to ensure that the reviewers were trained in the following:

- The use of the implicit review instrument.
- Referring consistently to certain pertinent sections of the case notes while conducting their reviews.
- Practicing reviewing notes for the purpose of this study and to discuss ambiguities with colleague reviewers and trainers in order to come to a common understanding of how difficult situations can be dealt with and what threshold others may place on quality and preventability.

### Statistical methods

**Figure 1:**
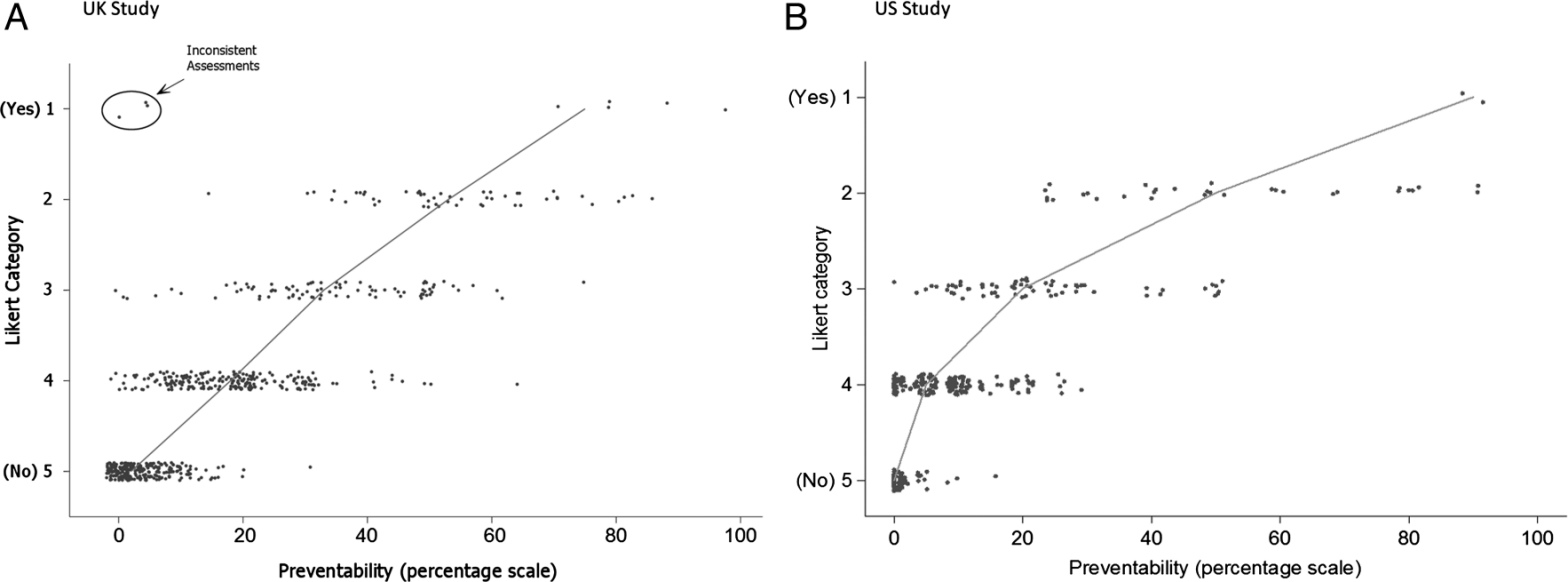
Association between the continuous (0–100) preventability scale and Likert categories. (A) UK study. (B) US study. The line connects the median preventability scores within each Likert category. Three points in the UK study have been circled as logically inconsistent and were removed from subsequent analyses.

The Likert and the continuous scale measurements were compared graphically (figure 1), and analysis of variance techniques (continuous scale by Likert category) were applied after excluding instances of logical inconsistency (ie, Likert 5 with preventability >50% or Likert 1 with preventability <50%). The calibration of the Likert scale with the continuous scale was described with a multilevel ordinal logistic regression analysis using a cubic polynomial function of the continuous scale with random coefficients at the reviewer level. The form of this polynomial was determined initially within a fixed effects ordinal logistic model. This polynomial function of the continuous scale was then used as an explanatory variable in a mixed effects version of the model with random slopes and intercepts.

Separately for each scale, the variance was decomposed into three components, corresponding to reviewers, case notes and a residual error term. This was done using a linear mixed effects model with (crossed) random effects for reviewers and case notes for the continuous scale data, and a corresponding mixed effects ordinal logistic model for the Likert data. The estimation of the case-note variance from this model defines the distribution of the latent variable (or latent scale) we are trying to measure. This latent scale represents the average reviewer's estimate of the probability of preventing death for each case (if the entire population of reviewers from which our reviewer sample was drawn had reviewed each record). Predictive distributions for the case-note effects on the Likert scale were derived by setting the reviewer and residual variances to zero in the ordinal logistic model, representing the predicted value for an ‘average’ reviewer, measured without error. Predictive distributions on the continuous scale were estimated in a similar way, from ordinal logistic models fitted to a categorised (representing 10 percentage points each) version of the continuous scale.

Models were fitted using the STATA V.14 and MLwiN V.2.28 packages.

## Results

### Data summary

#### UK study

Assessments were collected for 191 case notes from 22 reviewers. Of the 653 reviews, there were 644 that provided assessments of preventability on the Likert or continuous scale. Both scales were completed in 628 of the returns. Reviewers returned between 10 and 78 assessments each (mean 29.7). Of the 191 case notes, 19 had two reviews, 101 had three reviews, 48 had four reviews, 19 had five reviews, 3 had six reviews and 1 had seven reviews. The average number of reviews per case note was 3.4.The five categories of Likert responses (L1–L5) are summarised in table 1.

**Table 1:** Frequency (%) of Likert scale responses

	Was patient death preventable? n (%)					
	Definitely yes (L1)	Probably yes (L2)	Uncertain (L3)	Probably not (L4)	Definitely not (L5)	Total
UK study	8 (1.3)	51 (8.0)	86 (13.5)	220 (34.6)	270 (42.5)	635 (100.0)
US study	2 (0.5)	31 (8.1)	65 (17.0)	192 (50.1)	93 (24.3)	383 (100.0)

There were 637 continuous preventability assessments in total. The mean preventability was 17.5%, but the distribution is positively skewed with a median of 10% (quartiles, 3%, 28%), and with a high proportion (17.7%) of zeroes (113/637).

#### US study

This earlier study was smaller with 111 case notes reviewed by 13 reviewers. There were 383 reviews with assessments of preventability on the Likert or continuous scale. Reviewers carried out between 20 and 80 reviews each (mean 29.4). Of the 111 cases, 49 had one review, 33 had two, 14 had between three and eleven, and 14 had twelve or more. The average number of reviews per case note was 3.5. The five categories of Likert responses (L1–L5) are summarised in table 1 and had relatively fewer patients in the ‘definitely not’ preventable category than the UK study (24% vs 42%).

The 383 continuous preventability assessments had a mean preventability of 12.4%, with a median of 5% (quartiles, 0%, 20%) and 34% zeroes (133/383).

#### Logical consistency test: both studies

There were no instances of reviewers choosing Likert 5 (definitely not preventable) and providing a best preventability estimate of >50%, but in the UK study, there were three cases of reviewers choosing Likert 1 (definitely preventable) and providing a best estimate of <50%. This originated from two reviewers, where Likert 1 was chosen alongside best estimates of 0%, 4% and 5% (figure 1A). These were excluded from all analyses, as consideration of other reviewers' responses for these case notes suggested that the inconsistency derived from (hopefully momentary) confusion about the meaning of the Likert scale.

### Objective 1: correspondence between scales

The observed association between the Likert and per cent assessments in the UK study, where both scales are available (n=628), is illustrated in figure 1A. Figure 1B shows the US associations for 383 reviews where, again, both scales were available. There is clearly a strong correspondence between the scales. If the three inconsistent points indicated in figure 1A are excluded, the Likert categories account for 74% of the variation in the continuous scale, rising to 76% after adjustment for reviewer effects in the UK study and 73% of the variation in the US study.

**Figure 2:**
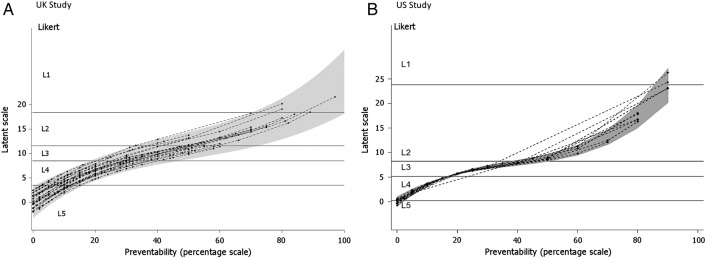
Estimated calibration plots for reviewers (22 UK; 13 USA) from a random slopes ordinal regression model. (A) UK study. (B) US study. A point on a curve represents the average latent score assessment given by an individual reviewer to a case note with a given percent preventability score. The shaded grey area defines a 95% prediction region over the population of reviewers for the mean latent score assigned to a given preventability percentage. The horizontal lines represent divisions on the latent scale corresponding to the Likert categories L1–L5.

Relatively large differences were found in the correspondence of the two scales between different UK reviewers using a multilevel ordinal logistic regression analysis which allows us to estimate how much the correspondence between the two measurements varies across reviewer. Figure 2A shows the best linear predictors of the calibration lines for the reviewers in the UK study, together with 95% prediction limits for calibration over the population of reviewers. A similar figure for the US study is shown in figure 2B. The variance components for the reviewers were smaller in magnitude (as seen in figure 2B).

### Objective 2: reliability of each scale

Cross-classified models (case note by reviewer) were fitted to the two scales, as described above. The proportion of the variance attributed to each component of variance is shown in table 2, estimated separately for each study. This represents the reliability of a single observation for estimating the true level of that component. For example, under the column ‘Case note’, we see the reliability of estimating the preventability of the death detailed in a particular case note using a single review by a randomly selected reviewer.

**Table 2:** Components of variance for preventability assessments, represented as proportions of the total variance for an individual assessment, with 95% CIs

Study	Scale	Case note	Reviewer	Residual error	Reliability of a single review
UK study	Likert	0.27 (0.19 to 0.39)	0.14 (0.05 to 0.34)	0.58 (0.44 to 0.71)	0.27
	Raw percentage	0.27 (0.19 to 0.36)	0.09 (0.04 to 0.21)	0.64 (0.54 to 0.73)	0.27
US study	Likert	0.23 (0.10 to 0.36)	0.17 (0.04 to 0.31)	0.60 (0.45 to 0.75)	0.23
	Raw percentage	0.22 (0.11 to 0.33)	0.07 (0.00 to 0.15)	0.71 (0.58 to 0.83)	0.22

The Likert variance decomposition refers to latent scales (the true score latent variable).

The residual error term represents an amalgam of two variance components: sampling variation (variation between repeat readings of a case note by the same reviewer) and the interaction between case notes and reviewers (the tendency for some case note/reviewer combinations to generate unexpected responses). Since no repeat readings were made with the same case note and reviewer, it is impossible to tease apart these components. The analyses on different scales—Likert and continuous—in both studies yield somewhat different results for the ‘reviewer’ components, but return similar estimates (∼27% for the UK study and ∼23% for the US study) for the reliability of a single review. Therefore, if a random reviewer was selected to review a random patient death from the UK sample, 27% of the variation in the resulting measurement would be due to signal (how preventable the death was) and 73% would be due to noise.

### Objective 3—impact of low reliability: extracting the case-note effect from the noise

The practical usefulness of the analysis depends on being able to describe the distribution of preventability scores across the case note sample, having removed the substantial amount of noise due to differences between reviewers on average across all their reviews (the reviewer component of variance in table 2) and the inconsistency from review to review (the residual error in table 2). This gives the estimates of the distribution of case-preventability ratings, conditional on the case notes all being reviewed by the reviewer most typical (the modal effect) of the reviewer population, and with the residual error term removed. The characteristics of these distributions could be used to compare samples of case notes arising from different times and institutions, if the reviewers are drawn from the same population.

**Figure 3:**
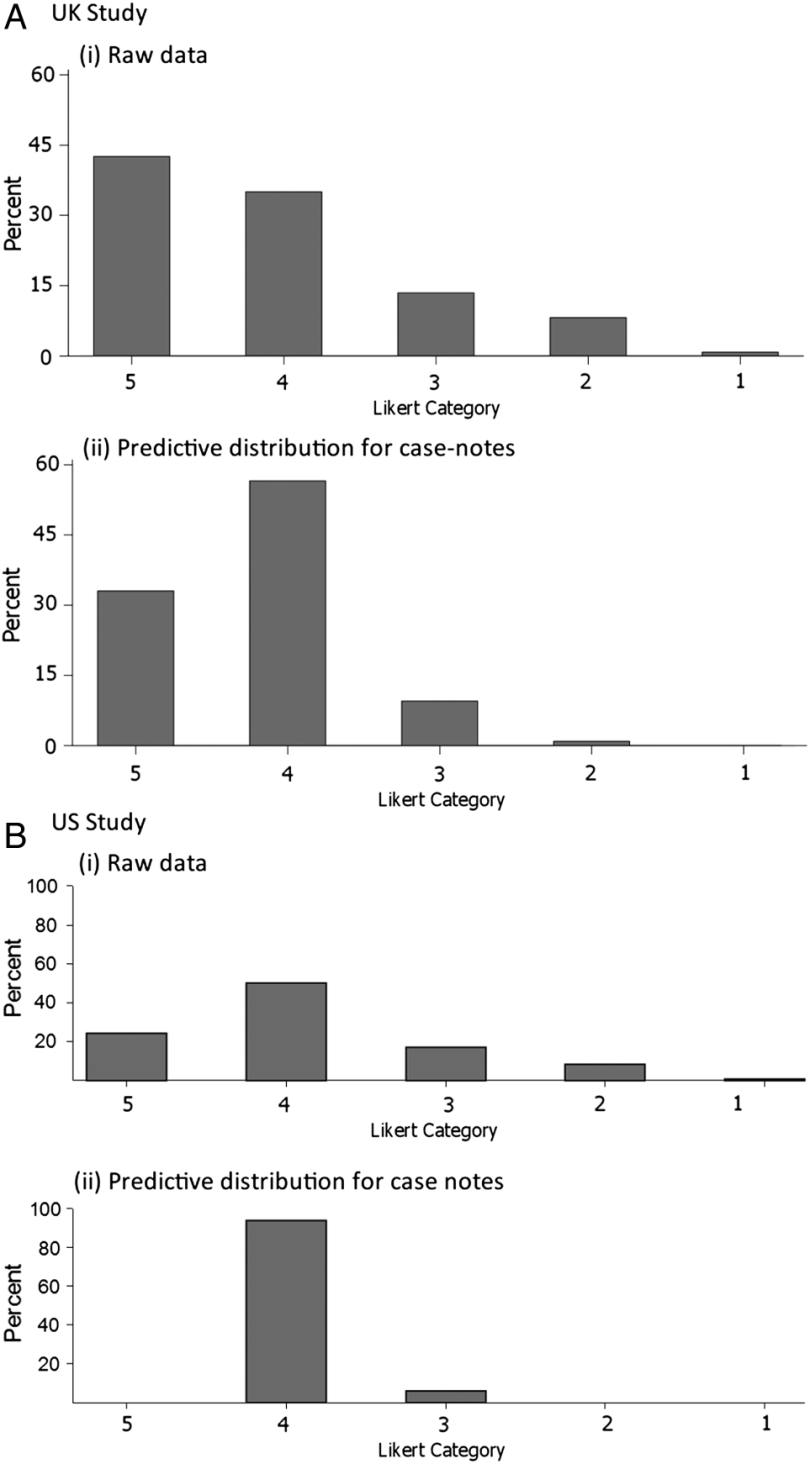
Likert category distributions: (i) raw data and (ii) predictive case note distribution after model-fitting. (A) UK study. (B) US study. For both distributions, the median lies in Likert category 4, that is, ‘probably not preventable’.

**Figure 4:**
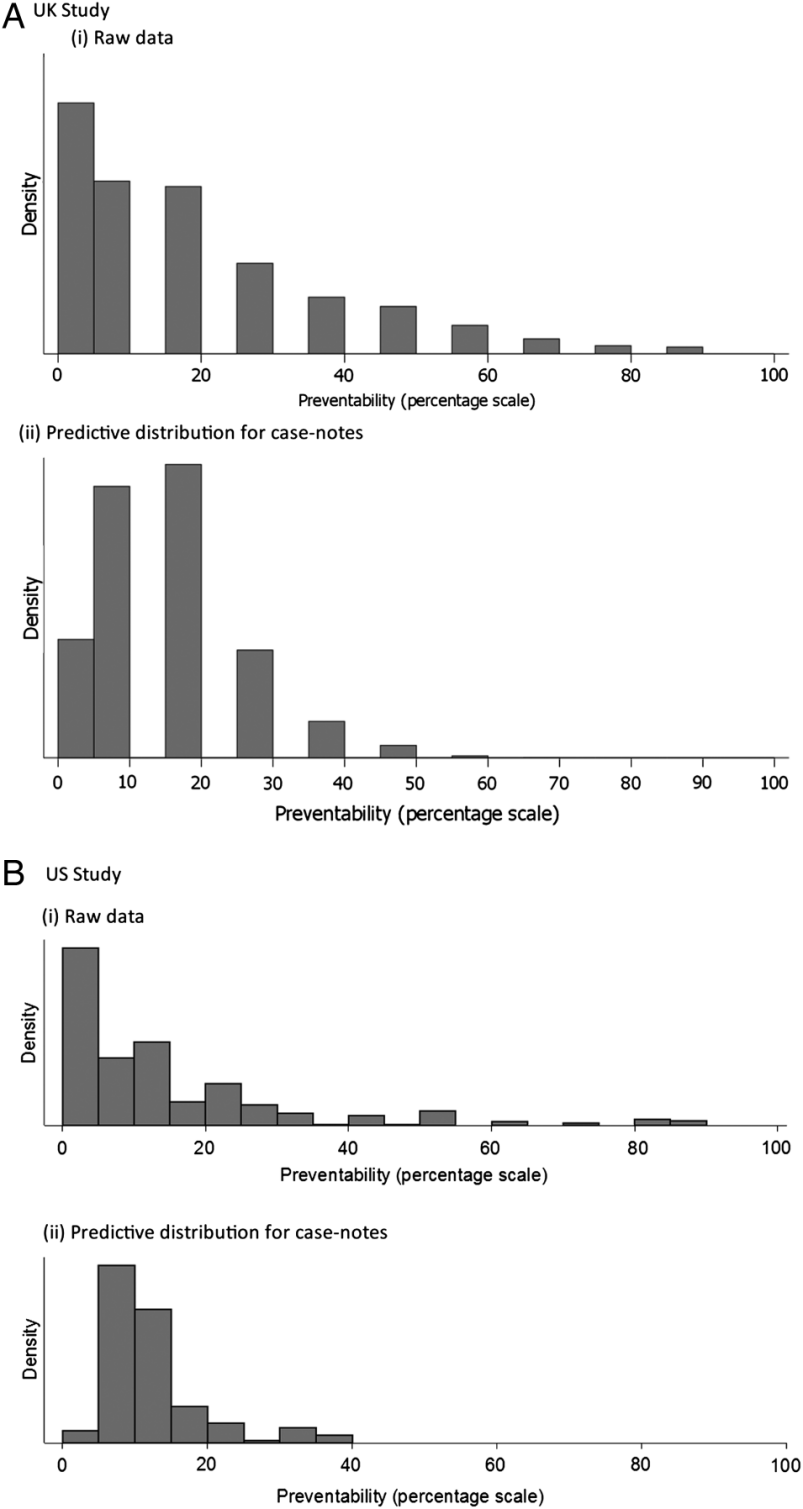
Distributions of percentage preventability: (i) raw data and (ii) predictive case note distribution after fitting a categorical model. (A) UK study. (B) US study.

Figures 3 and 4 show the predictive distribution of case note preventability across the categories used in the Likert analysis (figure 3) and a categorised version of the percentage analysis (figure 4). The charts in figures 3 and 4 represent the distribution of preventability (with the reviewer effects removed) among the population of case notes from which the sample is drawn.

In both cases, the predictive case note distribution is less extreme (ie, suggests fewer cases with high preventability) than the distribution of the raw data. The wider distribution in the raw data reflects lack of consistency across reviewers and reviews. For example, in the UK study, the median percentage preventability from the predictive distribution in figure 4Ai is estimated as 11.0%, which is similar to that for the raw data (10.0%), but the quartiles (7.0, 17.9) cover a much reduced range compared with the raw data (3.0, 28.0). Relevant to the standard of causation that the death was more likely than not to be preventable, after removing reviewer variation and measurement error, there were almost no case notes estimated as having more than uncertain likelihood of having patient death preventable by better quality of care in either the UK or US study (figure 3) when measured on the Likert scale, and few if any where the median reviewer would conclude that the ‘likelihood of prevention of death…if care had been optimal’ would exceed 50% (figure 4Aii, Bii).

## Discussion

### Main findings

In this paper, we have discussed some of the measurement characteristics of methods to judge the preventability of hospital deaths. In reference to our first objective, despite the two samples being very different in time period, country and design of the sample, the Likert scale and the continuous scale appear to behave in a similar fashion (figure 2). The reviewers appear to stop assigning the ‘uncertain preventability’ category and start assigning the ‘possibly’ and ‘probably preventable’ categories at just about exactly when they estimate the preventability on a continuous scale exceeds 50%. If the goal is to determine whether an average reviewer would feel that the death was more likely than not to be preventable, the observed correspondence provides support for grouping the response of ‘uncertain’ on a 5-point Likert scale along with the ‘possibly not’ and ‘probably not preventable’ responses.

In terms of our second objective, we find that the reliability of the Likert and continuous scale measurements were similar to each other in both datasets (0.27 vs 0.27 for the UK study; 0.23 vs 0.22 for the US study), suggesting no particular preference for one scale or the other in terms of precision. These low estimates of reliability, at about 0.2–0.3, are also consistent with almost all prior studies using expert review to estimate preventable deaths, quality of care or preventability of adverse events.^21^

Relating to our third objective, we show that the low reliability has considerable impact on drawing conclusions about the burden of preventable deaths, both at the individual and population level when using the ‘more probable than not’ standard of causation. To make a judgement at the individual level about a specific case, a reliability of 0.25 for a single measurement implies a need to average 12 independent reviews to achieve a reliability of 0.8 for a decision about whether any given death was preventable.^22^ ^23^ However, for an estimate at the hospital or system level, one can average across cases as well as reviewers, and reasonably precise estimates could be made with more practical numbers of reviews per case and total cases. In addition, as seen in figures 3 and 4, analyses that do not remove the noise or reviewer differences will significantly overestimate the degree to which reviewers think deaths are preventable.^10^ ^24^

### Limitations and strengths

The study was constrained by the original datasets. The Likert scale was always completed before the continuous scale on the case note review forms, whereas, ideally, the order would be randomised to mitigate practice effects. There were (small) differences in the precise descriptions given for the Likert categories in the UK and USA.

The strength of our study relies in its generalisability, given the different datasets in terms of time, place and clinical conditions. We were able to tease apart reviewer effects and residual errors in describing preventability across the case note sample. We carefully eliminated from the statistical analysis the few cases of ‘incoherence’—the provision of logically inconsistent answers.

### Implications

Relating to our fourth objective, what are the implications of our findings for the design of a programme that would attempt to measure the burden of preventable deaths in a health system? Our findings suggest that a Likert scale can reasonably represent expert opinion about causality, and there are no clear advantages in terms of precision to a continuous scale. However, the low reliability would suggest the need for a detailed consideration as to how to design the measurement procedure to find the optimal number of reviews per patient and independent reviewers needed to generate the estimates that the programme is supposed to produce at the required precision. Furthermore, to allow monitoring of the reliability of measurement and the estimates adjusted for that reliability, both reviewers and the case notes that they review must be more or less randomly distributed. This would preclude the use of reviewers only from the hospital that provides the cases, and require standardising the selection and training of reviewers across the health system.

However, it is crucial to point out that the interpretation of these numbers elicited from physician reviewers remains open to question. We would argue strongly against the interpretation that the measurements represent an objective probability that can be used to estimate a casualty count. It is critical to point out that there is really no evidence suggesting that this scale, elicited from physician experts by either of the two measurements, is anything more than ordinal with respect to the true probability of preventing death. Counterfactual reasoning is notoriously difficult, and there is evidence that physicians are not very good at estimating absolute prognostic probabilities and systematically increase their estimates of poor care when there is a bad outcome.^25^ ^26^

If we can only assume that the measurement is ordinal with respect to the true probability of death, then it makes no sense to dichotomise it and consider the cases on one side of the cut-off as ‘truly’ preventable and those on the other as not. In fact, it is extraordinary that this measurement procedure, which has been used in numerous studies, has been assumed to represent what would have to be a ratio scale of measurement, with a true zero and equal intervals for one to be able to estimate the actual burden of preventable deaths, in the absence of any evidence supporting that inference.

There may be a better solution. The measurement properties demonstrate that physicians, with a low, but not insignificant, level of reproducibility, can distinguish between patient case notes on a scale that is elicited by asking them to estimate the probability that a death could have been averted by optimal care. Why not give up on trying, after the fact, to estimate the probability of preventing death? It is, after all, a hubristic endeavour. Rather, ask the reviewers to estimate simply how good the care was in the cases of people who have died. This would not allow health systems to count up the number of deaths attributable to poor care, as they all seem to want to do. However, with a sufficient investment in a robust measurement system, it could allow systems to track relative performance across both hospitals and time, ensuring that attention could be focused on laggards and improvement of the system overall could be tracked. In that sense, review of deaths is really just a review of case notes enriched, one may suppose, to contain a higher proportion of serious errors. Review of deaths also enables doctors to be involved in quality assurance and to detect specific ‘bear traps’ to which they and others can be alerted.
