# Supplementary material for: Reviewing deaths in British and US hospitals: a study of two scales for assessing preventability
Source: BMJ Qual Saf. 2016 Jun 22;26(5):408–16. doi: 10.1136/bmjqs-2015-004849 (PMC5530333; doi:10.1136/bmjqs-2015-004849)
Supplement: Supplementary appendix [file bmjqs-2015-004849supp001.pdf]

Appendix 1: *Pro-formas* Used to Review Case Notes from the UK Study and the US Study.

[UK Study]

Semi-structured Implicit Pro forma for Collection of Data on Preventability of Mortality in Acute Medical Care in West Midlands Hospitals

Section 1 - Background

i. Case Note **Ref Number**: ______________

ii. Date of **Review**: ______________

iii. Date of **Admission**: ______________

iv. Date of **Death**: ______________

v. **Initials** of Reviewer: ______________

vi. Patient's **Age**: ______________

vii. Patient's **Sex**: ______________

Section 2 – Review of the notes

1a. **Primary** working diagnoses following admission in the **case-notes**:

____

(*Take diagnosis from the ward round in the first 48 hours post-admission when a ward round seems to have established a coherent management plan. If these do not exist take information from admission notes and tick here:*☐)

1b. **Primary** diagnosis on admission according to the **reviewer's opinion**:

☐ Agree with the above;

☐ Partially agree with the above;

☐ Do not agree with the above;

Please explain: ____

1c. **Final Primary** diagnosis after **establishment of all results**:

____

1d. **Final Primary** diagnosis after establishment of all results according to the **reviewer's opinion**:

☐ Agree with the above;

☐ Partially agree with the above;

☐ Do not agree with the above;

Please explain: ____

2a. **Known active co-morbidities** on admission according to the **case-notes**:

____

____

____

2b. **Known active co-morbidities** on admission according to the **reviewer's opinion**:

☐ Agree with the above;

☐ Partially agree with the above;

☐ Do not agree with the above;

Please explain: ____

3a. **New** diagnoses that post-dated admission, according to the **case-notes**:

____

____

____

3b. **New** diagnoses that post-dated admission, according to the **reviewer's opinion**:

☐ Agree with the above;

☐ Partially agree with the above;

☐ Do not agree with the above;

Please explain: ____

4a. **Primary** cause of **death** according to the last entry in the **notes** of doctor for cause of death:

☐ a) GP letter - explain ____

____

☐ b) Post Mortem - explain ____

____

☐ c) Last entry in case-notes - explain ________________________________

____

☐ d) Not given

4b. **Primary** cause of **death** according to **reviewer's opinion:**

☐ Agree with the above; if not given in the notes, please give your view:

____

☐ Partially agree with the above;

☐ Do not agree with the above;

Please explain: ____

5. **Please score the following**:

5a. Were there **clear management plans throughout** the admission?:

☐ 1. Definitely yes

☐ 2. Probably yes

☐ 3. Uncertain

☐ 4. Probably not

☐ 5. Definitely not

Please explain: ____

____

____

5b. **Appropriateness** of **diagnostic** work-up (investigations) for patient’s problem(s) throughout the admission:

- ☐ 1. Definitely appropriate
- ☐ 2. Probably appropriate
- ☐ 3. Uncertain
- ☐ 4. Probably not appropriate
- ☐ 5. Definitely not appropriate

Please explain: ____

____

____

5c. **Timeliness** of **diagnostic** procedures and evaluation for patient’s problem(s) throughout the admission:

- ☐ 1. Definitely timely
- ☐ 2. Probably timely
- ☐ 3. Uncertain
- ☐ 4. Probably not timely
- ☐ 5. Definitely not timely

Please explain: ____

____

____

5d. **Appropriateness** of **doctor’s orders** (management and treatment) for patient’s problem(s) throughout the admission, including inappropriate care / omissions / poor response to new diagnostic results:

- ☐ 1. Definitely appropriate
- ☐ 2. Probably appropriate
- ☐ 3. Uncertain
- ☐ 4. Probably not appropriate
- ☐ 5. Definitely not appropriate

Please explain: ____

____

____

5e. **Timeliness** of **doctors giving their management and treatment orders** for patient's problem(s) throughout the admission, including delayed orders, delayed response to diagnostic results and delayed speciality referral visits:

☐ 1. Definitely timely

☐ 2. Probably timely

☐ 3. Uncertain

☐ 4. Probably not timely

☐ 5. Definitely not timely

Please explain: ____

____

____

5f. **Errors in following** doctor's orders (timeliness and appropriateness), including administering medicines or fluids, or physician notification throughout the admission:

☐ 1. Definitely no errors

☐ 2. Probably no errors

☐ 3. Uncertain

☐ 4. Probably yes, had errors

☐ 5. Definitely yes, had errors

Please explain: ____

____

____

6. Please note any **adverse events** that were **<u>not</u>** the result of **poor care** (expected adverse events, for example due to expected side effects of drugs or procedures):

6a. ______________________________

6b. ______________________________

6c. ______________________________

6d. ______________________________

7. Please note any **adverse events** that **<u>might have been</u>** the result of **poor care**, including apparent omissions and episodes of sub-standard care in the following areas.

   8a. Diagnostic results either not acted upon, or acted upon after a delay;

   8b. Delay in care / inappropriate care / omission of care / poor response to direction of care;

   8c. Lack of plan for care / unclear direction of care;

   8d. Medication error (omissions and commissions, including fluid errors).

<u>Adverse Event</u>	<u>Error that caused it</u>
______________________	______________________
______________________	______________________
______________________	______________________
______________________	______________________
______________________	______________________
______________________	______________________

8. Rate the **quality of notes** and their **completeness** <u>in terms of your ability to extract the information required to fill this form</u> (*please chose one*):

☐ 1. Definitely adequate

☐ 2. Probably adequate

☐ 3. Uncertain

☐ 4. Probably not adequate

☐ 5. Definitely not adequate

Please explain:

____

____

____

____

9. Please give a very brief synopsis of this admission and events before death:

____

____

____

____

____

____

____

____

10. Overall how would you **rank** this **patients care** during this admission on a ten point scale (*please put a clear vertical line on the line where you intend to mark*)?

………
…….

0	1	2	3	4	5	6	7	8	9	10
(Very bad)					(Adequate)					(Very good)

11. Do you have any **further comments**? (Please write any other major concerns in care that did not lead to a particular adverse event, or is described above in other sections).

____

____

____

____

12. On the balance of probability (>50% chance), was the patient's death **preventable** through better quality of care during this admission?

☐ 1. Definitely yes

☐ 2. Probably yes

☐ 3. Uncertain

☐ 4. Probably not

☐ 5. Definitely not

Please comment:

____

13. Indicate on the following scale your **best estimate** of the **likelihood of preventability of death**, if care in this admission had been optimal (*please put a clear vertical line on the scale where you intend to mark*):

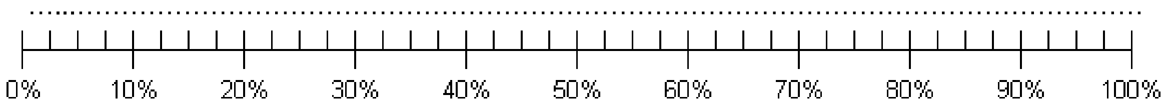

<u>**If you choose 25% or less, then please go to question 16.**</u>

14. Could this patient have **returned to their original functionality** before this episode of illness causing the admission, if **optimum care** was offered (**no errors** in care were made) and patient **had not died**?:

☐ 1. Definitely yes

☐ 2. Probably yes

☐ 3. Uncertain

☐ 4. Probably not

☐ 5. Definitely not

15. Please give your **best estimate** of **how long the patient would have survived** if optimum care was given (no errors in care were made) and patient had not died?:

☐ < 7 days ☐ 7 – 30 days ☐ 1 – 6 months ☐ 6 – 12 months ☐ > 12 months

Section 3 – Quality Control

16. How **long did it take** you to review this record and complete the questions?

____ minutes

Further Notes

____

____

____

____

____

____

____

____

1. Background Information

a) Patient key: **«KEY»**

~~b) Chart ID:~~

c) Admission Date: **«ADMDATE»**

d) Discharge Date: **«DISDATE»**

e) Age: **«AGE»** years

f) Sex: **«SEX»**

g) Discharge Status: **«DISPTYPE»** (*1-5 Alive, 6-7 Dead*)

2. Residence before Admission

a)
1. Home
2. Board & Care
3. Nursing Home
4. Relatives/Friends
5. Uncertain
6. Other b)______________________

3. DNR

a)
1. No
2. Yes, specify on which hospital day: b) ______________

c) Did an hospital complication or major decline occur prior to DNR status? 1. No 2. Yes

4. Inpatient Errors in Management

a) **Timeliness** of diagnostic evaluation for presenting problem(s)?
1. Definitely timely
2. Probably timely
3. Uncertain
4. Probably not timely
5. Definitely not timely

b) **Appropriateness** of diagnostic work-up for presenting problem(s)?
1. Definitely appropriate
2. Probably appropriate
3. Uncertain
4. Probably not appropriate
5. Definitely not appropriate

c) **Timeliness** of Initiation of treatment and obtaining consultation for presenting problem(s)?

1. Definitely timely
2. Probably timely
3. Uncertain
4. Probably not timely
5. Definitely not timely

d) **Appropriateness** of doctor's orders for patient's presenting problems?

1. Definitely appropriate
2. Probably appropriate
3. Uncertain
4. Probably not appropriate
5. Definitely not appropriate

e) Errors In following doctors' orders, administering medicines, or physician notification?

1. Definitely yes
2. Probably yes
3. Uncertain
4. Probably not
5. Definitely not

f) Appropriateness of physicians' response to new In-hospital signs, symptoms, or test results?

1. Definitely appropriate
2. Probably appropriate
3. Uncertain
4. Probably not appropriate
5. Definitely not appropriate

g) Procedural/Technical Adverse Outcomes?

1. Major adverse outcome What procedure? ______________________________
2. Moderate adverse outcome ______________________________
3. Minor adverse outcome What adverse outcome? ______________________________
4. No adverse outcome ______________________________

5. Preventable ?

a) Was patient death preventable by better quality of care?

1. Definitely yes
2. Probably yes
3. Uncertain
4. Probably not
5. Definitely not

b) Please comment:

____

____

____

c) What do you estimate the likelihood of prevention of death to be if care had been optimal? _______%

If patient had recovered from his/her acute/presenting illness (as well as could be expected), and had received optimal care, what would you rate as the probability that he/she would;

d) 1. Have left the hospital alive _______%

e) 2. Lived three months or more _______%

f) 3. Lived six months or more _______%

g) 4. Lived one year or more _______%

h) 5. Lived five years or more _______%

If patient had recovered from his/her acute/presenting illness/complication (as well as could be expected), and had received optimal care, what do you think would be the probability of the patient returning to:

i) 1. Good physical functioning _______%

j) 2. Good cognitive functioning _______%

6. Adequacy of Documentation in Medical Record

a) 1. Definitely adequate
2. Probably adequate
3. Uncertain
4. Probably not adequate
5. Definitely not adequate

7. Overall Quality of Medical Care

a) 1. Superior
2. Excellent
3. Good
4. Borderline
5. Substandard
6. Poor

b) If you answered 4-6, please specify reasons:

____

____

____

8.

a) _______________ b) ____________________________________ c) ____/____/____

Reviewer Initials | Estimated time (in minutes) for this review | Date
